# Determination of hand grip strength and its correlates during pregnancy: a cross-sectional study

**DOI:** 10.1186/s12884-021-04003-0

**Published:** 2021-08-04

**Authors:** Auwal Abdullahi, Amina Shuaib Bala, Sani Musa Danazumi, Saadatu Maiwada Abubakar, Rislanu Isyaku Adamu, Steven Truijen, Musa Kani Zakari, Christopher Olusanjo Akosile, Wim Saeys, Isa Usman Lawal, Mohammed Etoom, Jibril Mohammed Nuhu, Mukadas Akindele Oyeniran, Kabir Isah Mayana, Ushotanefe Useh

**Affiliations:** 1 Department of Physiotherapy, Bayero University, Kano, Nigeria; 2 Department of Rehabilitation Sciences and Physiotherapy, University of Antwerp, Movant, Wilrijk Belgium; 3 Department of Physiotherapy, Federal Medical Center, Nguru, Yobe State Nigeria; 4 Department of Physiotherapy, University of Maiduguri Teaching Hospital, Maiduguri, Nigeria; 5 Department of Medical Rehabilitation, Nnamdi Azikiwe University, Awka, Nigeria; 6 Department of Physiotherapy, Aqba University of Technology, Aqaba, Jordan; 7 Lifestyle Diseases Research Entity, Faculty of Health Sciences, North-West University, Potchefstroom, South Africa; 8 Faculty of Health Sciences, University of Salford, Salford, UK

**Keywords:** Pregnancy, Blood pressure, Body mass index, Gravidity, Hand grip strength, Physical function, Breastfeeding, Activities of daily living

## Abstract

**Background:**

Pregnancy results in many changes, including reduced hand grip strength (HGS). However, good HGS is required for physical functions such as carrying and breastfeeding the baby after birth. The aim of this study was to determine the factors that may predict HGS during pregnancy.

**Methods:**

The study was a cross-sectional study approved by the Research Ethics Committees of Kano State Ministry of Health and Aminu Kano Teaching Hospital in Kano, north-west, Nigeria. Pregnant women at the designated hospitals were included in the study if they had no serious comorbidities or any known neurological condition that affects the hands and the neck. Demographic characteristics and independent (predictor) variables (age, weight, height, BMI, maternity leave status, number of full-term deliveries, number of preterm deliveries, number of live births, number of abortuses, gravidity, trimester, systolic blood pressure, diastolic blood pressure, inter arm systolic BP difference [IASBP], inter arm diastolic BP difference [IADBP], and heart rate) of each of the participants were recorded by experienced therapists. The data were analysed using descriptive statistics, t-test, Pearson correlation coefficient and standard multiple regression.

**Result:**

One hundred and sixty-one pregnant women with mean age, 25.04 ± 4.83 years participated in the study. In the dominant hand, 120 participants (74.5%) had weak grip strength. In the non-dominant hand, 135 participants (83.9%) had weak grip strength. For the dominant hand, the total variance explained by the whole model was significant, 28.5%, F(11, 161) = 1.187, R^2^ = 0.081, p = 0.300 . In the final model, none of the variables significantly predicted HGS. However, systolic blood pressure contributed to the model more than any other variable (Beta = -0.155). For the non-dominant hand, the total variance explained by the whole model was not significant, 33.1%, F(11, 161) = 1.675, R^2^ = 0.111, p = 0.089 . In the final model, only systolic blood pressure (Beta = -0.254, p = 0.023) significantly predicted hand grip strength.

**Conclusion:**

Cardiovascular events or changes during pregnancy (such as change in systolic blood pressure) may be related to HGS in pregnant women. It is therefore, important for clinicians to pay attention to this, in planning rehabilitation strategies for pregnant women.

**Supplementary Information:**

The online version contains supplementary material available at 10.1186/s12884-021-04003-0.

## Introduction

Pregnancy can cause physiological, psychological and physical changes in women. Some of the physical changes include musculoskeletal changes such as reduced hand grip strength (HGS) [1, 2]. Reduced HGS during pregnancy is believed to be caused by several factors including hormonal changes (such as high level of circulating oestrogen), altered nutritional status and increased protein level (which may result in fluid retention in the body, including the wrist) [3, 4]. In particular, a high level of circulating oestrogen and increased protein level can result in fluid retention, which occurs in up to 17% to 62% of pregnant women [3–6]. When there is fluid retention in the upper extremities, carpal tunnel syndrome in which the median nerve is compressed may result, which may in turn, result in reduced HGS [3, 7].

Reduced HGS may help indicate health outcomes. This is because hand grip strength is an indicator of many health outcomes such as physical strength, cognition, functional status, mobility, pulmonary function, and cardiovascular health [2, 8–11]. Consequently, reduced HGS has been linked to poor muscle mass, decreased walking speed and physical activity level, as well as increased risk of death due to cardiovascular diseases [9, 12–16]. In addition, HGS was reported to have a strong correlation with various anthropometric characteristics in both pregnant and non-pregnant women [2, 17, 18]. Furthermore, HGS can be affected by demographic factors such as age and gender [8]. Similarly, it may also be related to ethnicity and culture [19].

Reduced HGS is characterised by pain, numbness, difficulty in grasping objects, muscle weakness and tendency of things to drop from the hand [6, 20]. However, pregnant women require good HGS to carry out their daily living activities such as eating, washing, writing and grooming. Likewise, they require good hand grip strength to carry their babies, bathe and breastfeed them after giving birth. According Wade and Taylor, weakness of the upper extremity following delivery, such as in the idiopathic postpartum brachial neuritis, may result in difficulty in carrying out daily activities such as washing one’s hair, pouring water from the jug and throwing a ball [21]. This report echoed the importance of upper limb function including HGS during pregnancy and after delivery. Unfortunately, routine check for HGS does not seem to be used during pregnancy in Kano, Nigeria. The aim of this study was to determine HGS and the factors that could predict it during pregnancy. In addition, the study aimed to look at the difference in HGS between trimesters. This is because knowing the status of HGS during pregnancy and the factors that could predict it can help clinicians in designing rehabilitation strategies to prevent or manage reduced HGS by addressing the predictors.

## Method

The study used a cross-sectional research design. The population of the study was pregnant women attending antenatal clinics at Aminu Kano Teaching Hospital (AKTH), Murtala Muhammad Specialists Hospital (MMSH) and Muhammad Abdullahi Wase Specialist Hospital (MAWSH), all in Kano, Nigeria. The study was approved by the Research Ethics Committees of AKTH (AKTH/MAC/SUB/12A/P-3/VI/2392) and Kano State Ministry of Health (MOH/Off/797/T.I/740). Participants were included if they were pregnant (no age limit was considered because, in the environment where the data was collected, women get married before the age, 18), and had neither movement restriction in the upper limbs nor positive history of any neurological disorder. The participants were consecutively recruited into the study. In addition, all participants provided written informed consent for their participation in the study.

The minimum sample size for the study was 143. This was calculated using G power software [22]. The calculation done was a priori for multiple linear regression, fixed-effect model. The parameters used were effect size (f^2^) = 0.15 (moderate effect size, alpha value = 0.05, power = 80% and number of predictors = 16 (age, weight, height, BMI, maternity leave status, number of full-term deliveries, number of preterm deliveries, number of live births, number of abortuses, gravidity, trimester, systolic blood pressure, diastolic blood pressure, inter arm systolic BP difference [IASBP], inter arm diastolic BP difference [IADBP], and heart rate). We included maternity leave status as one of the independent variables because it may affect the participants’ level of physical activity, which may also affect HGS. Data for this study was collected using a proforma for demographic and other independent variables, a portable weighing scale for body weight, tape measure (60 inches/150 cm, Shanghai, China) for height, Camry electronic hand dynamometer (EH101, United Kingdom) for HGS and sphygmomanometer, and stethoscope (Litman, USA) for blood pressure. An electronic hand dynamometer is an affordable and a reliable instrument for measuring of hand grip strength [23, 24].

Body weight was measured with the participants in minimal clothing and without shoes. The measurement was carried out in kg to the nearest 0.5 kg using a portable weighing scale [25]. Height was measured with the subjects standing erect and bare-footed against a calibrated wall with their feet together on a level floor. A horizontal ruler was rested on their heads, and the height was read from the wall [26]. The measurement was also carried out in centimetres to the nearest 0.5 cm, and converted to meters thereafter. Body Mass Index (BMI) was calculated as weight in kilogram divided by height in meter squares [27]. BMI values were categorized as underweight = BMI below 18.5 kg/m^2^, normal weight = BMI from 18.5 kg/m^2^ to 24.9 kg/m^2^, over weight = BMI from 25 kg/m^2^ to 29.9 kg/m^2^ and obese = BMI above 30.0 kg/m^2^ [27].

For the measurement of HGS, the participants were seated comfortably in a chair with the arm resting on the arm rest. They were then made to hold the test arm of the dynamometer at a 90° elbow flexion, with the forearm placed in a neutral position and the hand parallel to the forearm. Thereafter, the participants were asked to squeeze the dynamometer to the best of their ability three times. This was carried out for both hands starting with the dominant hand, followed by the non-dominant with two minutes interval between them. The measurements were recorded in kilograms and the mean of the three trials was taken as the measure of  HGS. The three trials were carried out with each of the hands with one minute rest period between them.

Similarly, to measure blood pressure (BP), the participants were seated comfortably in a chair with the arm resting on the arm rest. The  BP was measured using a mercury sphygmomanometer and was undertaken twice on each arm (for both the dominant and non-dominant hands). One-minute interval was allowed between each measurement. We chose one minute interval because blood pressure can change with time. Therefore, we decided on a short interval. Following the measurements, the average was obtained and used as the BP value. All measurements were carried out in a conducive and well ventilated environment to avoid overestimating or underestimating the measurement since temperature can affect blood pressure. In addition, an appropriate cuff size was used, and the correct placement of the cuff was achieved (with lower edge of the cuff about 1 inch above elbow crease and the cuff’s bladder over the brachial artery) in order to obtain accurate readings. The diaphragm of the stethoscope was placed over the brachial artery. The participants were also instructed to relax and avoid any thought or activity that could raise their level of anxiety prior to the measurement. Data at the three study sites were collected by experienced physiotherapists (one in each study site) who were blinded from the aim of the study. The data collection took place between 4^th^ June, 2018 and 30^th^ August, 2018.

The data obtained were analysed using Statistical Package for Social Sciences (SPSS) version 20.0 and a p-value of < 0.05 was considered significant in the analysis. The normality of the data distribution was assessed using Kolmogrov-Smirnov statistics which showed normal data distribution (p > 0.05). Consequently, parametric statistics (Pearson product moment correlation) was used to analyse the relationship between the independent and dependent variables; and between the independent variables. In addition, the difference in HGS between trimesters was analysed using analysis of variance (ANOVA). The difference between trimesters was determined using ANOVA because the measurement was carried out once in each trimesters. Therefore, the rationale was to determine the variance between trimesters especially that there were differences in the number of participants between trimesters. Furthermore, descriptive statistics of mean, frequency, distribution tables, and standard deviation were used to describe the data.

Finally, standard multiple regression analysis was carried out to determine whether the independent variables age, BMI, maternity leave status, number of abortuses, number of live births, number of termed deliveries, number of preterm deliveries, trimester, heart rate, systolic BP, diastolic BP, inter arm systolic BP difference and inter arm diastolic BP difference could predict HGS in pregnant women. Standard multiple regression analysis means the independent variables were entered into the model to help determine the variable or variables that predict HGS better or more than the others without considering the order of entry of the variables into the model.

## Results

**Fig. 1:**
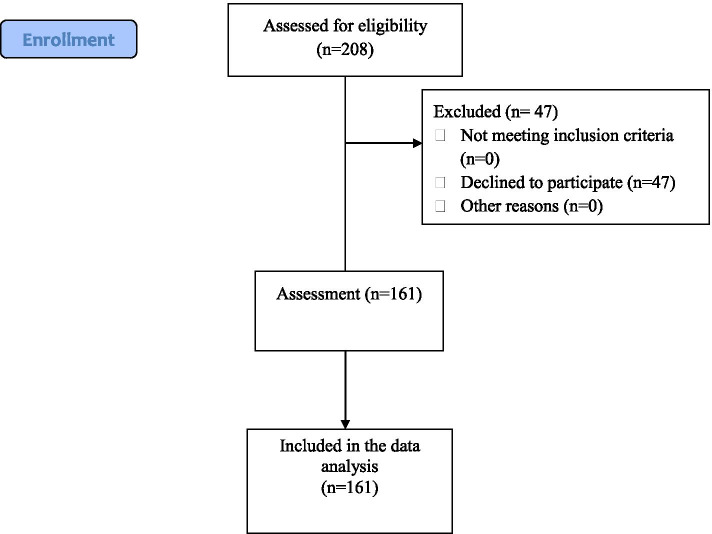
The Study Flowchart

A total of 161 pregnant women with age range, 17 to 39 years participated in the study (see Fig. 1 for the study flowchart). Details of the characteristics of the study participants are presented in Table 1.

**Table 1:** Characteristics of the Study Participants (*n* = 161)

Variable	Mean ± SD	Frequency	Percentage (%)
Age	25.04 ± 4.83 years		
Age category			
17–22 years	55	34.2	
23–28 years	68	42.2	
29–34 years	30	18.6	
35–40 years	8	5	
Weight	62.38 ± 11.53 kg		
Height	2.49 ± 12.02 m		
BMI	26.28 ± 5.12 kg/m^2^		
Ethnicity Y/H/I/F/K		4/114/8/30/5	2.5/70.8/5.0/18.6/3.1
Maternity leave Yes/No/NA		6/63/92	3.7/39.1/57.1
Occupation A/B/H/C/S		7/35/95/12/12	4.3/21.7/59.0/7.5/7.5
Education N/P/S/T/None		9/11/91/49/1	5.6/6.8/56.5/30.4/6
Socioeconomic L/M/H		4/156/1	2.5/96.9/6
Marital status S/M		3/158	1.9/98.1
Trimester 1st/2nd/3rd		19/50/92	11.8/31.1/57.1
History of Hypertension Yes/No		18/143	11.2/88.8
Gravidity	3.06 ± 2.10		
SBP	115.23 ± 10.27 mmHg		
DBP	74.76 ± 8.93 mmHg		
ISBP	4.59 ± 5.51 mmHg		
IDBP	1.42 ± 3.53 mmHg		
Heart Rate	83.86 ± 12.03 beats/min		
HGS (d)	20.46 ± 4.61 kg		
HGS (nd)	7.87 ± 4.40 kg		

KEY:*Y/H/I/F/K = Yoruba/Hausa/Igbo/Fulani/Kanuri,*NA = Not Applicable, *A/B/H/C/S = Artisan/Business/House wife/Civil servant/Student,* N/P/S/T/None =  Non-formal /Primary/Secondary/Tertiary/None, *L/M/H = Low/Middle/High, *S/M = Single/Married, *SBP = Systolic Blood Pressure, *DBP = Diastolic Blood Pressure, *IADSBP = Inter Arm Difference in Systolic Blood Pressure, *IADDBP = Inter Arm Difference in Diastolic Blood Pressure, *HGS (d) = Hand Grip Strength dominant,*HGS (nd) = Hand Grip Strength non dominant

In the dominant hand, 120 participants (74.5%) had weak grip strength. In the non-dominant hand,  135 participants (83.9%) had weak grip strength. Overall, the dominant and non-dominant hands’ mean HGS was 20.46 ± 4.61 kg and 7.87 ± 4.40 kg respectively, indicating reduced grip strength. The normal values of HGS for the left and right hand are 30.8 (27.2 to 34.5) kg and 33.8 (29.5 to 38.1) kg respectively [28].

The result also showed no significant difference (p > 0.05) in grip strength between trimesters. The result is presented in Table 2.

**Table 2:** Differences in grip strength across trimesters (*n* = 161)

Variable	Trimester				
	First (*n* = 19)	Second (*n* = 50)	Third (*n* = 92)		
	Mean ± SD			F	P-value
Dominant hand	21.53 ± 5.16	20.22 ± 4.24	20.37 ± 4.70	0.596	0.552
Non-dominant hand	19.77 ± 3.58	17.21 ± 3.87	17.84 ± 4.73	2.383	0.096

For both the dominant and non-dominant hands, HGS is correlated with the independent variables. See Tables 3 and 4 for the dominant and non-dominant hands respectively. For the independent (predictor) variables, full-term deliveries and live births (r = 0.936, p < 0.001), and full-term deliveries and gravidity (r = 0.930, p < 0.001) were highly correlated. See Table 5 for the details of the result of correlation analysis between the independent variables. Similarly, BMI is a variable gotten from the variables, weight and height. According to Pallant, when two independent variables are highly correlated (r = 0.9 and above); or when an independent variable is a product of two different independent variables, such variables should be excluded from multiple regression analysis to avoid violation of assumptions of multicollinearity and singularity respectively [29]. Consequently, the variables, full-termed deliveries, live births, gravidity, weight and height were removed from the analysis.

**Table 3:** Relationship between dominant HGS with the clinical variable of the participants (n = 161)

Dependent variable	Independent variable	Correlation(r)	p-value	
Handgrip strength	Age	0.128	0.106	
	Weight	0.141	0.075	
	Height	0.007	0.933	
	BMI	0.006	0.943	
	Maternity leave	-0.116	0.142	
	Termed deliveries	0.017	0.835	
	Pretermed deliveries	-0.023	0.775	
	Abortuses	-0.113	0.155	
	Livebirth	0.036	0.652	
	Gravidity	0.037	0.641	
	Trimester	-0.055	0.485	
	Systolic BP	0.101	0.200	
	Diastolic BP	0.053	0.508	
	IADSBP	0.103	0.192	
	IADDBP	0.079	0.318	
	Heart Rate	0.070	0.376	

KEY: *HGS*  Hand Grip Strength, *BMI * Body Mass Index, *BP* Blood pressure, *IADSBP* Inter Arm Difference in Systolic Blood Pressure, *IADDBP* Inter Arm Difference in Diastolic Blood Pressure

^*^Significant at p ≤ 0.05

**Table 4:** Relationship between non dominant HGS with the clinical variable of the participants (n = 161)

Dependent variable	Independent variable	Correlation(r)	p-value
Handgrip strength	Age	0.155	0.050
	Weight	0.110	0.165
	Height	0.028	0.725
	BMI	0.78	0.328
	Maternity leave	-0.120	0.131
	Termed deliveries	0.014	0.855
	Pretermed deliveries	-0.040	0.616
	Abortuses	- 0.138	0.081
	Livebirth	0.039	0.624
	Gravidity	-0.046	0.559
	Trimester	-0.080	0.314
	Systolic BP	0.137	0.082
	IADSBP	0.012	0.876
	IADDBP	-0.004	0.963
	Heart Rate	-0.029	0.712

KEY: *HGS* Hand Grip Strength, *BMI * Body Mass Index, *BP* Blood pressure, *IADSBP* Inter Arm Difference in Systolic Blood Pressure, *IADDBP*  Inter Arm Difference in Diastolic Blood Pressure

^*^Significant at *p* ≤ 0.05

**Table 5:** Relationship between the Independent Variables (n = 161)

VARIABLES	Age	Weight	Height	BMI	Maternity leave	Full--term deliveries	Preterm deliveries	Abortuses	Live birth	Gravidity	Trimester	SBP	DBP	IADSBP	IADDBP	Heart rate
Age																
Weight	r=0.329 p<0.001															
Height	r=0.150 p=0.058	r=0.154 p=0.051														
BMI	r=0.269 p=0.001	r=0.875 p<0.001	r=-0.303 p<0.001													
Maternity leave	r=-0.138 p=0.082	r=-0.069 p=0.382	r=-0. 171 p=0.030	r=0.020 p=0.802												
Full-term deliveries	r=0.630, p<0.001	r=0.104 p=0.190	r=0. 071 p=0.371	r=0.104 p=0.191	r=-0.081 p=0.310											
Preterm deliveries	r=-0.068 p=0.394	r=-0.078 p=0.324	r=0. 010 p=0.889	r=-0. 083 p=0.297	r=0.061 p=0.445	r=-0.144 p=0.068										
Abortuses	r=0.080, p=0.314	r=0.011 p=0.891	r=0.008 p=0.921	r=0.039 p=0.625	r=0.062 p=0.432	r=0.155 p=0.050	r=-0.075 p=0.343									
Live births	r=0.607, p<0.001	r=0.116 p=0.143	r=0.04 p=0.550	r=0.114 p=0.150	r=-0.051 p=0.158	r=0.936 p<0.001	r=-0.112 p=0.156	r=0.165 p=0.036								
Gravidity	r=0.571, p<0.001	r=0.097 p=0.222	r=0.061 p=0.440	r=0.109 p=0.170	r=-0.033 p=0.676	r=0.930 p<0.001	r=-0.081 p=0.306	r=0.490 p<0.001	r=0.881, p<0.001							
Trimester	r=0.029, p=0.712	r=0.127 p=0.110	r=-0.143 p=0.070	r=0.198 p=0.102	r=-0.031 p=0.676	r=0.082 p=0.302	r=0.125 p=0.113	r=0.095 p=0.230	r=0.078, p=0.322	r=0.117 p=0.138						
SBP	r=0.124, p=0.117	r=0.279 p<0.001	r=0.029 p=0.713	r=0.242 p=0.02	r=-0.209 p=0.008	r=-0.034 p=0.673	r=-0.082 p=0.304	r=-0.117 p=0.139	r=-0.024, p=0.445	r=-0.096 p=0.226	r=-0.625 p=0.413					
DBP	r=0.061, p=0.442	r=0.127 p=0.029	r=0.072 p=0.364	r=0.141 p=0.073	r=-0.060 p=0.447	r=-0.070 p=0.375	r=-0.040 p=0.610	r=0.046 p=0.563	r=-0.043, p=0.588	r=-0.063 p=0.428	r=0.021 p=0.794	r=0.629 p<0.001				
IADSBP	r=-0.021, p=0.792	r=-0.069 p=0.437	r=0.083 p=0.297	r=-0.089 p=0.259	r=0.008 p=0.915	r=-0.018 p=0.820	r=0.085 p=0.286	r=0.066 p=0.405	r=-0.061, p=0.445	r=0.007 p=0.929	r=0.229 p=0.004	r=0.084 p=0.292	r=0.076 p=0.336			
IADDBP	r=0.096, p=0.228	r=-0.033 p=0.680	r=0.130 p=0.101	r=-0.089 p=0.263	r=-0.203 p=0.010	r=-0.051 p=0.517	r=0.049 p=0.538	r=0.054 p=0.494	r=-0.062, p=0.432	r=-0.025 p=0.756	r=0.053 p=0.508	r=0.069 p=0.384	r=-0.027 p=0.737	r=0.317 p<0.001		
Heart rate	r=0.038, p=0.635	r=-0.109 p=0.167	r=0.073 p=0.357	r=-0.127 p=0.109	r=-0.109 p=0.170	r=0.154 p=0.051	r=-0.118 p=0.136	r=-0.105 p=0.184	r=0.129, p=0.103	r=0.071 p=0.369	r=-0.041 p=0.602	r=0.025 p=0.753	r=0.022 p=0.780	r=-0.088 p=0.265	r=-0.083 p=0.293	

**Fig. 2:**
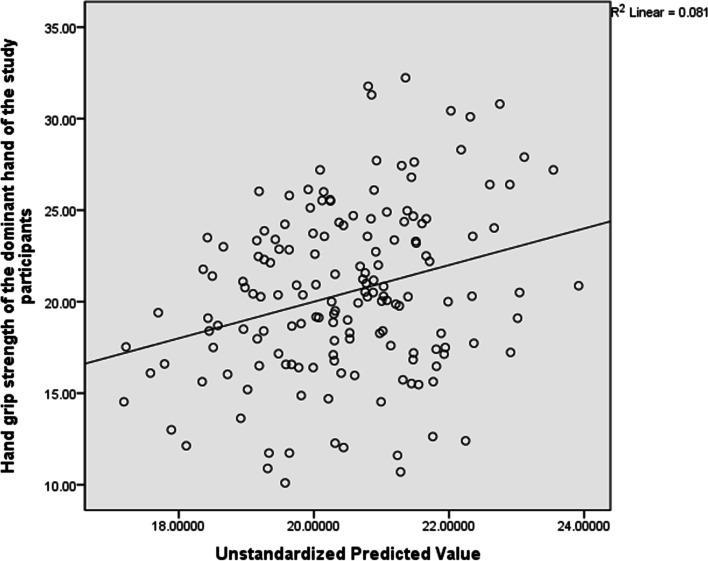
Scatter Plot for the Predictors of Hand grip strength in the dominant hand indicating the regression line

For the dominant hand, the total variance explained by the whole model was not significant, 28.5%, F(11, 161) = 1.187, R^2^ = 0.081, p = 0.300 . In the final model, none of the variables significantly predicted HGS. However, systolic blood pressure contributed to the model more than any other variable (Beta = -0.155). See Table 6 and Fig. 2 for the details of this analysis.

**Table 6:** Predictors of Hand Grip Strength in the dominant hand (*n* = 161)

Variables	B	r	95% CI	*P*-value
Age	0.129	0.129	-0.030 to 0.288	0.112
BMI	-0.049	0.005	-0.207 to 0.109	0.540
Maternity leave	-0.673	-0.117	-2.022 to 0.676	0.326
Preterm deliveries	-0.591	-0.023	-5.342 to 4.160	0.806
Abortuses	-0.582	-0.113	-1.475 to 0.310	0.199
Trimester	-0.529	-0.055	-1.692 to 0.572	0.344
SBP	0.069	0.162	-0.030 to 0.169	0.170
DBP	-0.021	0.053	-0.129 to 0.087	0.702
IADSBP	0.070	0.088	0.072 to 0.212	0.330
IADDBP	-0.045	0.040	-0.268 to 0.177	0.687
Heart rate	-0.047	-0.070	-0.110 to 0.016	0.146

*BMI* Body Mass Index, *SBP* Systolic Blood pressure, *DBP* Diastolic Blood Pressure, *IADSBP* Inter Arm Difference in Systolic Blood Pressure, *IADDBP* Inter Arm Difference in Diastolic Blood Pressure

^*^Significant at *p* ≤ 0.05

**Fig. 3:**
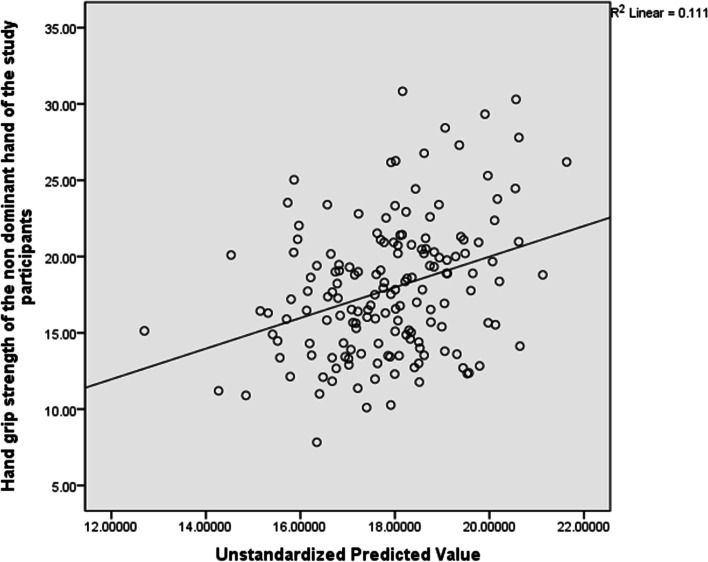
Scatter Plot for the Predictors of Hand grip strength in the Non-dominant hand indicating the regression line

For the non-dominant hand, the total variance explained by the whole model was not significant, 33.1%, F(11, 161) = 1.675, R^2^ = 0.111, p = 0.089 . In the final model, only systolic blood pressure (Beta = -0.254, p = 0.023) significantly predicted hand grip strength. See Table 7 and Fig. 3 for the details of this analysis.

**Table 7:** Predictors of Hand Grip Strength in the non-dominant hand (*n* = 161)

Variables	B	r	95% CI	*P*-value
Age	0.128	0.152	-0.021 to 0.277	0.091
BMI	0.002	0.078	-0.146 to 0.149	0.983
Maternity leave	-0.662	-0.120	-1.924 to 0.600	0.302
Preterm deliveries	-0.219	-0.040	-4.678 to 4.239	0.923
Abortuses	-0.521	-0.138	-1.357 to 0.315	0.220
Trimester	-0.173	-0.080	-1.741 to 0.316	0.173
SBP	0.109	0.191	0.016 to 0.202	0.023*
DBP	-0.073	0.027	-0.175 to 0.028	0.154
IADSBP	-0.018	- 0.033	-0.151 to 0.115	0.790
IADDBP	-0.134	-0.062	-0.343 to 0.075	0.207
Heart rate	-0.035	-0.029	-0.094 to 0.024	0.248

*BMI*  Body Mass Index, *SBP* Systolic Blood pressure, *DBP* Diastolic Blood Pressure, *IADSBP* Inter Arm Difference in Systolic Blood Pressure, *IADDBP* Inter Arm Difference in Diastolic Blood Pressure

^*^Significant at *p* ≤ 0.05

**Fig. 4:**
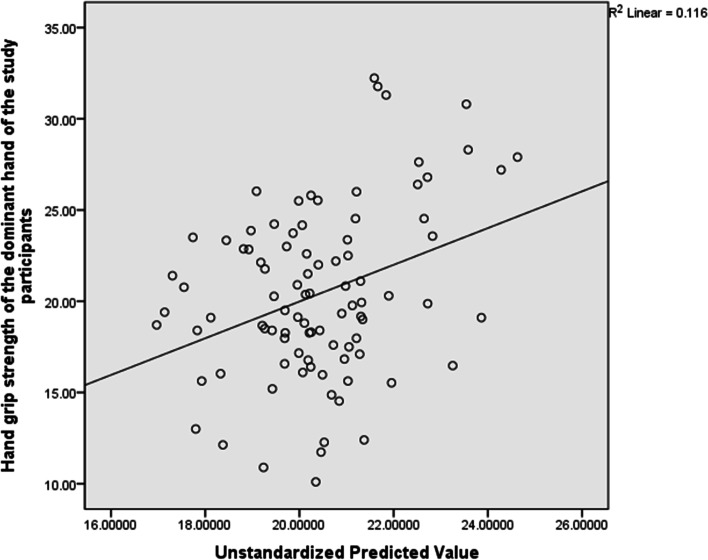
Scatter Plot for the Predictors of Hand grip strength in the dominant hand of participants in the 3^rd^ trimester indicating the regression line

Although, there was no significant difference in HGS between trimesters, but majority of the participants (n = 92) were within the 3^rd^ trimester. Therefore, we conducted a posthoc analysis to determine whether age, BMI, maternity leave status, number of preterm deliveries, number of abortuses, systolic blood pressure, diastolic blood pressure, inter arm systolic BP difference (IASBP), inter arm diastolic BP difference (IADBP), and heart rate will significantly predict HGS during the 3^rd^ trimester. The result showed that, for the dominant hand, the total variance explained by the whole model was not significant, 34.0%, F(10, 92) = 1.060, R^2^ = 0.116, p = 0.402 . In the final model, only systolic blood pressure (Beta = 0.323, p = 0.034) significantly predicted HGS. See Table 8 and Fig. 4 for the details of this analysis.

**Table 8:** Predictors of Hand Grip Strength in the dominant hand for participants in the 3^rd^ trimester (n = 92)

Variables	B	r	95% CI	*P*-value
Age	0.135	0.129	-0.104 to 0.374	0.625
BMI	-0.052	0.047	-0.266 to 0.163	0.632
Maternity leave	0.020	-0.095	-1.767 to 2.079	0.983
Preterm deliveries	-0.604	-0.027	-5.584 to 4.375	0.810
Abortuses	-0.637	-0.152	-1.770 to 0.496	0.267
SBP	0.148	0.207	0.011 to 0.286	0.034*
DBP	-0.113	-0.018	-0.266 to 0.041	0.148
IADSBP	0.110	0.115	-0.101 to 0.321	0.302
IADDBP	-0.124	0.052	-0.433 to 0.185	0.428
Heart rate	-0.039	-0.029	-0.129 to 0.052	0.400

*BMI* Body Mass Index, *SBP* Systolic Blood pressure, *DBP* Diastolic Blood Pressure, *IADSBP* Inter Arm Difference in Systolic Blood Pressure, *IADDBP* Inter Arm Difference in Diastolic Blood Pressure

^*^Significant at *p* ≤ 0.05

**Fig. 5:**
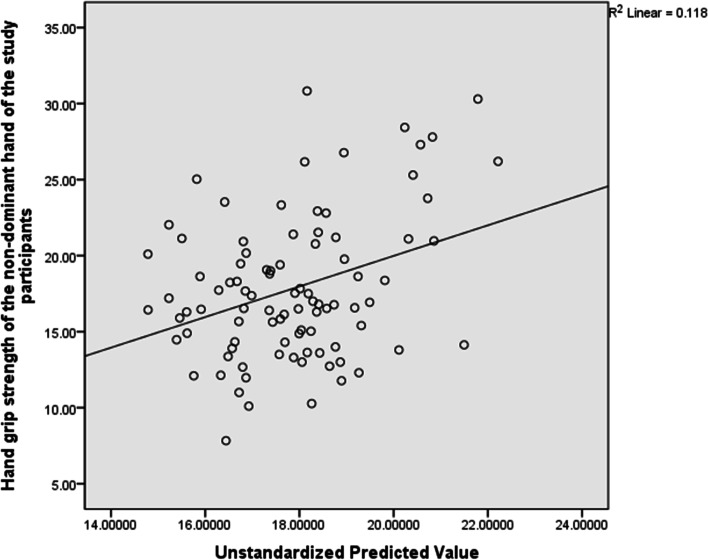
Scatter Plot for the Predictors of Hand grip strength in the non-dominant hand of participants on the 3^rd^ trimester indicating the regression line

For the non-dominant hand, the total variance explained by the whole model was not significant, 34.1%, F(10, 92) = 1.068, R^2^ = 0.116, p = 0.396 . In the final model, none of the independent variables significantly predicted HGS. See Table 9 and Fig. 5 for the details of this analysis.

**Table 9:** Predictors of Hand grip strength in the non-dominant hand for participants in the 3^rd^ trimester (*n* = 92)

Variables	B	r	95% CI	*P*-value
Age	0.169	0.182	-0.072 to 0.409	0.166
BMI	-0.027	0.091	-0.243 to 0.189	0.804
Maternity leave	-0.376	-0.131	-2.255 to 1.503	0.692
Preterm deliveries	-0.965	-0.048	-5.971 to 4.041	0.702
Abortuses	-0.724	-0.152	-1.863 to 0.415	0.210
SBP	0.114	0.217	-0.024 to 0.252	0.105
DBP	-0.054	0.051	-0. 208 to 1.00	0.489
IADSBP 0.049	0.042	-0.163 to 0.261	0.648	
IADDBP	-0.106	0.054	-0.417 to 0.206	0.502
Heart rate	-0.063	-0.079	-0.155 to 0.028	0.170

*BMI* Body Mass Index, *SBP* Systolic Blood pressure, *DBP* Diastolic Blood Pressure, *IADSBP* Inter Arm Difference in Systolic Blood Pressure, *IADDBP* Inter Arm Difference in Diastolic Blood Pressure

^*^Significant at *p* ≤ 0.05

## Discussion

The main aim of this study was to determine HGS and its predictors in pregnant women. The result of the study showed that only systolic blood pressure significantly predicted HGS. Both reduced grip strength and increased blood pressure have been reported to be caused by hormonal changes, increased protein level and fluid retention in pregnant women [2, 30, 31]. One of the mechanisms of reduced HGS is carpal tunnel syndrome due to median nerve compression [3, 32]. However, aerobic exercise has been reported to normalise hormonal changes and protein level, and reduce fluid retention during pregnancy [33]. Even moderate to high intensity exercises are considered safe during normal pregnancies, and provide immense benefits for both maternal and foetal health such as maternal glycaemic control and uteroplacental and foetal blood flow, foetal growth, and increased muscle strength [34–38].

Similarly, it may help reduce fatigue and deconditioning during pregnancy and after delivery [39–41]. Therefore, it is important that pregnant women are prescribed regular aerobic exercise to help prevent or combat reduced HGS. This is because HGS and general strength of the upper limb are required for breastfeeding and caregiving of the baby after birth. In the absence of adequate hand grip and upper limb strength, these may not be possible, and the families may have to involve volunteer or paid caregivers. Considering how many times babies need to be breastfed per day, this can result in caregiver burden or huge financial loss on the volunteer caregiver and the paid caregiver allowance. However, strenuous exercises that can jeopardize the lives of the mother and the foetus such as a long jump should be avoided. In addition, if pregnant women developed reduced HGS that extended to after delivery, measures such as the use of adaptation pillow can be used to offer some support during breasting or holding the baby [42]. Upper limb functional activities such as opening jars and carrying bags during pregnancy could also help influence HGS. As such, these types of activities should be encouraged during pregnancy to help prevent reduced HGS.

In addition, in the present study, the result showed no significant difference in HGS between trimesters. However, in a recent study, it was reported that, weak grip strength was more prevalent in the third trimester [43]. This may not be unconnected to the fact that, during the third trimester, there is usually increased levels of hormones, especially oestrogen, which is implicated in the pathophysiology of reduced HGS [1, 44]. Incidentally, most of the participants of the present study were within the third trimester of pregnancy. Therefore, one of the reasons for the difference could be because of the ethnic difference between the participants of the present study and the ones in the previous studies. Oestrogen level varies across ethnic groups [19]. Secondly, the participants in the present study (25.04 ± 4.83 years) are younger as indicated by their mean age compared to the ones in the previous study (29.57 ± 3.43 years). Consequently, they might not have started experiencing a natural decline in  HGS. According to Angst and colleagues, natural decline in  HGS starts after the age 40 years [45]. This could explain why in the present study, there was no significant correlation between age and HGS. Thirdly, the lack of difference between trimesters could be due to an unequal number of participants in the three trimesters.

In a previous study, HGS was shown to have no significant association with blood pressure in the elderly; but it has a significant association with the middle-aged participants [46]. Although the aforementioned study was on apparently healthy individuals, the present study also found no significant correlation between HGS and blood pressure (both diastolic and systolic). Thus, age could be an important factor for HGS [45]. Interestingly, the present study found that systolic blood pressure is a statistically significant predictor of HGS among other clinical and socio-demographic variables in the non-dominant hand. However, when only the data of the participants in the 3^rd^ trimester was analysed, systolic blood pressure also significantly predicted HGS in the dominant hand . Long standing high systolic blood pressure could probably lead to inefficiency in the pumping of blood by the heart, which will ultimately lead to fluid retention. Fluid retention may worsen the pathophysiology of reduced HGS [3, 7]. Additionally, heart rate increases when the heart has to pump blood with great effort [47]. Consequently, there was a negative and a weak, but non-significant correlation between heart rate and HGS in the present study. Thus, high blood pressure and increased heart rate may likely reduce HGS in pregnant women. In contrast, it was reported in studies on apparently healthy individuals that increased blood pressure is associated with increased HGS [14, 46]. The difference could be because, in pregnancy, hormonal changes also contribute to decrease in HGS [30].

Another variable in the literature that is related to HGS is BMI, and is said to exist regardless of hand dominance [48, 49]. Similarly, in the present study, a strong but non-significant relationship between BMI and HGS in the non-dominant hand was found. When the BMI is high, it may also be associated with increased risk of high blood pressure [14]. According to the findings of this study, high blood pressure in particular, the systolic pressure is related to decrease in HGS. Therefore, those with high BMI may likely have reduced  HGS. In addition, the higher the number of gravidity a woman had, and as she grows older, she might have increased level of oestrogen and decreased  HGS[45]. Increased level of oestrogen is associated with reduced HGS [30]. Consequently, in the present study, gravidity was reported to have a weak positive but non-significant relationship with  HGS which indicates that, the higher the gravidity, the more likely for pregnant women to have reduced HGS due to physical and physiological changes that result from pregnancy. Furthermore, it was stated that increased number of parity influences BMI which causes body adiposity [50, 51]. Level of adiposity also significantly influences HGS in females [2].

Full-term deliveries appear to be the best predictor of HGS even though it is not significant. This indicates that the more gravidity and the more pregnancy is to term, the more physiological changes occur in the body, leading to symptoms that cause reduced HGS. However, this study has some limitations or weaknesses. One of its weaknesses is the lack of use of qualitative inquiry to explore the pregnant women’s experience with their HGS during pregnancy. In addition, lack of assessment of mobile phone use during pregnancy, and the level of physical activity of the study participants were some of the other potential weaknesses of this study. This is because, mobile phone usage, and physical activity level can affect HGS [15, 52]. Moreover, the research design itself (cross-sectional study) is also a potential limitation since the findings can only explain the relationship between the dependent and the independent variables, not cause and effect. Another additional limitation may be the lack of age-matched control and assessment of participants for carpal tunnel syndrome to actually confirm that the reduced HGS is due to pregnancy or related symptoms. In contrast, some of the strengths of this study are its relatively large sample size, and the provision of data from an ethnic, national or racial group not previously available and the possibility of highlighting it as a possible consideration for physical therapists and other professionals in medical rehabilitation in this environment.

## Conclusion

Cardiovascular events or changes during pregnancy (such as change in systolic blood pressure) may be related to HGS in pregnant women. Therefore, it is important for clinicians to monitor pregnant women with reduced HGS for fluctuations in systolic blood pressure or vice-versa. This will help them design novel exercises or rehabilitation strategies that can be used to combat the effects of reduced HGS and high blood pressure capable of endangering the lives of the expectant mother and the foetus or the baby after birth. In addition, future studies should determine how long post-delivery does it take for a person to regain  HGS; and whether strength and conditioning exercise protocol is beneficial post-delivery.

## Supplementary Information

**Additional file 1.**

## Data Availability

The data for this study is available on request to the corresponding author
